# Early Transcriptional Signature in Dendritic Cells and the Induction of Protective T Cell Responses Upon Immunization With VLPs Containing TLR Ligands—A Role for CCL2

**DOI:** 10.3389/fimmu.2019.01679

**Published:** 2019-08-02

**Authors:** Ariane C. Gomes, Mona O. Mohsen, Julius E. Mueller, Fabiana M. S. Leoratti, Gustavo Cabral-Miranda, Martin F. Bachmann

**Affiliations:** ^1^The Jenner Institute, Oxford University, Oxford, United Kingdom; ^2^Immunology, Inselspital, Bern, Switzerland

**Keywords:** VLP (virus-like particle), CpG—oligonucleotides, dendritic cell (DC), T cell—DC interactions, CCL2

## Abstract

Inducing T cell responses by therapeutic vaccination requires appropriate activation of antigen presenting cells (APCs). The use of virus-like particles (VLPs) containing Toll-like receptor (TLR) ligands has demonstrated remarkable potential in activating APCs and modulating the immune response both for prophylactic vaccines as well as immunotherapy. Here, we employed VLPs associated to TLR ligands as tools to modulate cytotoxic response mediated by CD8^+^ T cells and provide further insight in the development of T cell-based immunotherapy. We have investigated the *in vivo* transcriptional signature in dendritic cells (DCs) from mice immunized with VLPs containing distinct classes of nucleic acid and correlated the expression patterns with the efficiency of induced T cell responses. We identified key pathways activated in DCs that are involved in the appropriated induction of T cell responses and show evidence for the modulatory effect of CCL2 in CD8^+^ T cells responses. These insights shed light on immune networks that are pivotal for the induction of potent cytotoxic T cell responses and identify key genes for appropriate DC activation and subsequent modulation of the adaptive immune response.

## Introduction

In spite of all prophylactic vaccines in use to date promoting protection through neutralizing antibodies, there is a growing interest in developing vaccines that induce protection mediated by T cells. Therapeutic vaccines against chronic viral infections and cancer in particular, seem to benefit from the induction of T cell responses, in particular cytotoxic responses mediated by CD8+ T lymphocytes (CTL) (1–3). However, unlike some vaccines that induce neutralizing antibody responses and have been developed empirically, induction of effective antigen-specific T cell responses has proved itself a complex endeavor that will require major developmental strides before reaching the market (4). Considering the distinct regulation and complexity of the activation events for B and T lymphocytes, it is reasonable to assume that unlike B cell inducing vaccines, attempts to develop T cell inducing vaccines will not be successful until we have a better understanding of the regulatory networks of T cell responses. T cell responses are tailored by the innate immune system through costimulatory and inhibitory molecules and cytokine/chemokine clues mostly promoted by antigen presenting cells (APCs), especially DCs (5). Appropriate activation of APCs is critical for successful vaccine development strategies since APCs are responsible not only for initiating but also sustaining the adaptive immune response. Therefore, understanding the factors that drive appropriate activation of APCs that correlate to the adaptive response becomes paramount for vaccine development, especially when aiming for T cell mediated protection.

The process of developing safer vaccines has in most cases rendered antigenic candidates less immunogenic. Thus, the search for an appropriate adjuvant—i.e., an adjuvant that will modulate the response toward the desired outcome—can be considered almost as important as the choice of the antigenic target during vaccine development. TLR ligands have emerged as a promising adjuvant for vaccines due to their safety and remarkable ability to enhance immunogenicity with efficacy already proven in the clinics (1, 6, 7). TLR7/8 and TLR9 ligands, such as ssRNA and CpG, especially in combination with delivery systems such as nanoparticles and VLPs, have been showing promising results in the induction of cytotoxic T cells and have been successfully employed in a plethora of pre-clinical vaccines for different indications. Understanding the initial events induced by TLR-ligands, in particular in DCs, can be a valuable tool for the development of effective adjuvants and therapeutic vaccines. To gain insight into these early events, we have employed systematic and integrative analysis of *in vivo* gene expression in DCs preceding three types of CD8^+^ T cell responses after vaccination: (i) absent vs. (ii) inefficient vs. (iii) protective T cell responses. We have previously demonstrated that VLPs containing B type CpG are able to induce protective T cell responses in tumor models caused by human papilloma virus (HPV) (8) and viral infections (9) while its VLP counterparts containing RNA were unable to provide protection against tumor growth and associated mortality. Using the same vaccine model, we have investigated the transcriptional signature in DCs the first day following immunization *in vivo* with these different vaccines and correlated the initial events and pathways to cross-priming of T cells by DCs.

## Materials and Methods

### VLPs, Vaccine Preparation and Immunizations

Qβ VLPs were prepared as previously described (8). Packaging of CpG B into Qβ VLPs was performed by digestion of bacterial RNA with RNAse A (Merck). RNAse-treated VLPs were either further purified to remove RNAse and degraded RNA to make VLPs devoid of nucleic acid [Qβ(o)] or combined with CpG. Excess of CpG and RNAse was removed by dyafiltration (Amicon). Packaging of CpG A G10 was performed by re-assembling as previously described (10). Re-packaging and integrity of VLPs were assessed by co-localization of nucleic acid and protein on a native agarose gel stained with SYBR safe (Invitrogen), followed by Coommasie blue stain (Expedon).

Vaccines were prepared by chemical coupling of antigens and VLPs using the heterobifunctional cross-linker succinimidyl-6-(β-maleimidopropionamido) hexanoate (SMPH, Pierce, USA) following manufacturer's instructions.

Type A CpG G10 were custom made by Eurogentec. Sequence: 5′gggggggggggacgatcgtcgggggggggg 3′. CpGs 1668 with phosphorothioate backbone were purchased from Invivogen (sequence: 5′ tccatgacgttcctgatgct 3′). Immunodominant peptides p33 was produced in a modified version with additional 3 aa (GGC) added to the C terminus (Proimmune, UK) to allow chemical crosslinking of peptide to VLPs. P33 peptide sequence: KAVYNFATMGGC.

### Mice

Wild-type C57BL/6 mice were purchased from Envigo (UK). CCL2 knockout mice were described previously (11) and purchased from Jacksons lab, USA. STING−/− mice was provided by John Cambier and it have been described elsewhere (12). MAVS−/− mice has been provided by Jan Rehwinkel and described elsewhere (13).

All mice used in this study were bred in specific pathogen-free (SPF) conditions. This study was carried out in accordance to the recommendations of the Animals (Scientific Procedures) Act 1986 (ASPA) and European Directive 2010/63/EU on the protection of animals used for scientific purposes. Protocols were approved by the Animal Welfare and Ethical Review Bodies at the Nuffield Department of Clinical Medicine, University of Oxford (PPL 30/2947).

### Cells Preparation and Sorting

Spleens were isolated at indicated time-points and prepared for analysis.

*Cell sorting—*Sorting of splenic DCs was performed with immunomagnetic positive selection of mouse CD11c+ cells (EasySep Mouse CD11c, StemCell Tech) according to the manufacture's instruction.

*Conventional bone-marrow derived DCs (BMDCs) and bone-marrow derived plasmacytoid DCs (BMpDCs) generation*—bone marrow cells were cultured in complete RPMI for 6–8 days in the presence of granulocyte-macrophage colony-stimulating factor (GM-CSF) containing X-63 cell supernatant for generation of conventional BMDCs or FLT3 (Insight Biotech) for generation of BMpDCs.

### Tetramer Staining and Cytokine Measurement

Total splenocytes of immunized mice were isolated 24 h following immunization and single-cell suspension was prepared as and cultured for further 12 h. Secreted cytokines (G-CSF, GM-CSF, IFN-γ, IL-1α, IL-1β, IL-2, IL-4, IL-5, IL-6, IL-7, IL-9 IL-10, IL-12p40, IL-12p70, IL-13, IL-17, CCL2, CCL3, CCL5, CXCL1, CXCL2, CXCL10) were measured with a Milliplex kit (Merk-Millipore, France) following the manufacturer's instructions. Luminex MAGPIX system with xPONENT software was used for data acquisition and data analysis. INF-α, IL-12p40/70, and IL-1α/β were measured by ELISA following manufacturer's instruction (Thermo Fisher, R&D, and R&D, respectively).

T cell response was assessed 7 days following immunization. For tetramer staining freshly isolated splenocytes were stained and immediately analyzed with anti-CD8 and p33 Tetramer. For cytokine production, cells were re-stimulated with p33 peptides.

### Antibodies and Flow Cytometry

Flow cytometry was performed using a BD FACSCanto II and the following antibodies:

APC-Cy7 anti-CD11c (N418, Invitrogen), PerCP-Cy5.5 anti-CD3, AlexaFluor700 anti-CD8, FITC anti-TNFα (eBioscience), APC anti-IFNγ (Life Tech), PE anti-CD101 (Thermo), PE anti-CD86 (eBioscience), Acqua Live-Dead dye (Thermo), FITC anti-CD40, APC conjugated Tetramer-p33_33−41_ (H-2D^b^ KAVYNFATC, NIH core tetramer facility) were used.

For intracellular staining of IFN-γ, IL-12, and TNF-α, splenocytes were fixed and permeabilized with BD Cytofix buffer. Antibody staining was performed subsequently to Fc receptor blockade (monoclonal antibody 2.4G2 to mouse CD16-CD32, 10 μg/ml) in PBS supplemented with 0.1% FBS.

Data analysis was performed in the FlowJo software (TreeStar).

### Immunofluorescence and Endosomal Trafficking of VLPs and Free CpG

Cells were cultured on glass-bottom, 0.17 mm tissue culture dishes (MatTek GLASS). BMDCs were incubated with CellLight^®^ Lysosomes-GFP following the manufacturer's instructions (Life Technologies). Qβ VLP were packaged with a type B CpG 1668 coupled to a fluorescent dye (custom made, Eurogentec). Confocal microscopy studies were performed with an oil immersion objective (63x oil immersion, numerical aperture 1.4) on Zeiss LSM 710 Multiphoton Confocal Microscope.

Type B CpG sequence and modifications: 5′ tccatgacgttcctgatgct 3′ coupled to AlexaFluor 547 dye. Packaging of CpG-AF547 was performed as described previously on this manuscript for unlabelled CpG.

### RNA Preparation, RNA-seq and Primary Data Analysis

*Sample preparation -* Total RNA was extracted from sorted splenic DCs from immunized mice following the manufacturer's protocol (RNeasy Midi Kit, Qiagen), using an on-column DNase-I digest step.

Material was quantified using RiboGreen (Invitrogen) on the FLUOstar OPTIMA plate reader (BMG Labtech) and the size profile and integrity analyzed on the 2,200 or 4,200 TapeStation (Agilent, RNA ScreenTape). Library preparation was completed using TruSeq Stranded Total RNA kit (Illumina) following manufacturer's instructions. Libraries were amplified on a Tetrad (Bio-Rad) using in-house unique dual indexing primers (14). Individual libraries were normalized using Qubit, and the size profile was analyzed on the 2,200 or 4,200 TapeStation. Individual libraries were normalized and pooled together accordingly. The pooled library was diluted to ~10 nM for storage. The 10 nM library was denatured and further diluted prior to loading on the sequencer. Paired end sequencing was performed using a HiSeq4,000 75bp platform (Illumina, HiSeq 3,000/4,000 PE Cluster Kit and 150 cycle SBS Kit).

*Data analysis -* All statistical and bioinformatics analysis was performed using R language (https://www.r-project.org) and Bioconductor packages.

Trimmomatic 0.35 package was used in order to quality filter reads and remove adapter contamination (15). The first 10 and the last base were removed after visual inspection of the read quality distribution. After trimming, read length was 64BP.

For read mapping, Star aligner v2.5.2b (16) was used on the Gencode M12 transcriptome (mm 10) with settings “–outSAMmultNmax 20.” Reads were assigned to genes using featureCounts v1.5.1 (17) with settings “-C -B -M -s 2 -p”. To calculate log2 fold change estimates between groups, a negative binomial general linear model was fit using DESeq2 (18) and only genes with a Benjamini-Hochberg (19) corrected Wald test FDR of less than 5% were labeled significant.

The overrepresentation of pathways within groups of DE genes was computed by applying a one-tailed Fisher's exact test. Only top-level pathways with *P* < 0.0001 for overrepresentation were considered. Plots were generated in R language with the package ggplot2.

Heatmaps were generated using counts per million multi mapped (CPM-MM) values as input. CPM-MM values are mean centered, row scaled and standardized. Hierarchical clustering was performed by using the distance based Euclidean distances using complete linkage clustering.

## Results

### Packaging of CpGs Into VLPs Alters Endosomal Trafficking and Immune Responses

**Figure 1:**
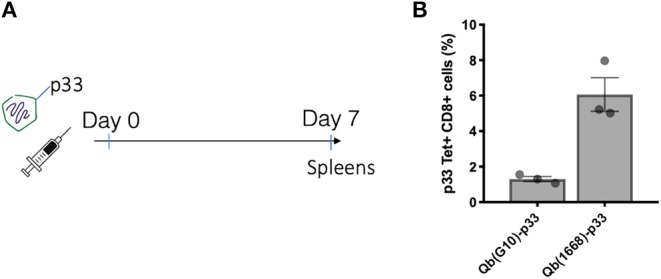
CpG B 1668 is superior than CpG A G10 at inducing CTL responses. **(A)** Immunization schedule. Mice were immunized s.c. with 50 μg of Qβ containing either G10 type A CpG (Qb(G10)-p33), or 1668 B type CpG Qβ(1668)-p33. Spleens were collected 7 days later and p33 antigen-specific CD8+ T cells were analyzed by **(B)** p33-specific Tetramer staining. Data represented as mean + SEM. *N* = 3 mice.

Considering the distinct immune responses induced by the three classes of CpG (20, 21) and the widespread use of CpG associated to nanoparticles, we wanted to evaluate the impact of packaging different classes of CpG—type A and type B—into VLPs in the context of T cell-inducing vaccines [for a review on different types of CpG see (6)]. Hereafter, VLPs packaging CpG will be represented by Qβ(G10) for type A CpG G10, and Qβ(1668) for type B CpG 1668, an schematic representation of re-packing process and quality assessment of re-packed VLPs are demonstrated on **Figures 3A,B**. To compare the ability of Qβ(G10) and Qβ(1668) to induce T cell responses, VLPs were cross-linked with the immunodominant model peptide p33, derived from Lymphocytic Choriomeningitis virus (LCMV). Mice were immunized and CTL responses assessed by tetramer staining 7 days later (Figure 1A). Mice receiving Qβ(1668)-p33 had at least 3-fold higher frequencies of p33-specific T cells compared to Qβ(G10) (Figure 1B).

**Figure 2:**
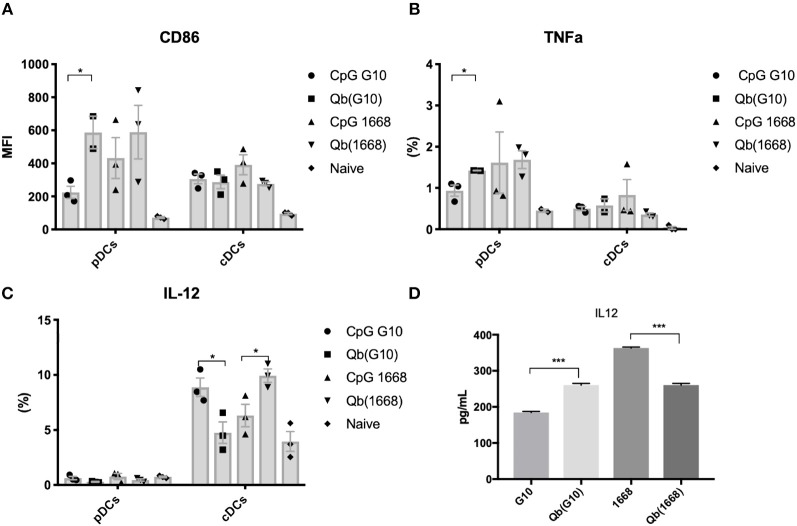
Packaging of CpGs on VLP alters the immune response induced by VLPs. **(A)** CD86 expression and **(B)** TNF-α production by splenic pDCs and cDCs. **(C)** IL-12 production by splenic cDCs (CD11c+CD317-) and pDCs (CD11c+CD317+) treated with 1 μM of the respective CpG or 100 μg/mL of VLP for 24 h. **(D)** IL-12 production by murine splenocytes incubated with either 1 μM of the free CpG or 100 μg/mL of the respective VLP for 24 h. Data represented as Mean + SEM, *n* = 3 mice for splenocytes and for BMDCs, pooled bone marrow of 2 mice with 3 biological replicates per condition. Statistical significance was measured by ANOVA followed by multiple *t-*tests followed by Bonferroni and Dunn's correction for multiple testing ^***^p < 0.001, ^*^p < 0.01.

To further investigate the differences on CTL responses generated by CpG A G10 vs. CpG B 1668, the initial activation of DCs induced by each CpG was evaluated. To that end, freshly isolated splenocytes or bone marrow derived pDCs and cDCs were stimulated for 24 h and activation markers and cytokines were measured in response to free CpGs and their packaged counterparts. The activation molecule CD86 was up-regulated in DCs in response to all preparations, however free type B CpG was found to be superior at inducing CD86 up-regulation especially in pDCs, where a more striking difference was observed (Figure 2A). TNF-α was produced mostly by pDCs and at higher levels by the cells treated with free type B CpG or the VLPs compared to type A CpG (Figure 2B). IL-12, a key cytokine associated with Th1 response and known to be induced at high levels by type B CpG was produced at different levels depending not only on the stimuli but also on the cell type being investigated (Figures 2C,D). cDCs produced higher levels of IL-12 in comparison to pDCs in response to all stimuli. When measuring IL-12 produced by total splenocytes (Figure 2D), a different trend was observed with higher levels of IL-12 being produced in response to free CpGs most likely due to B cells that are also express TLR9.

**Figure 3:**
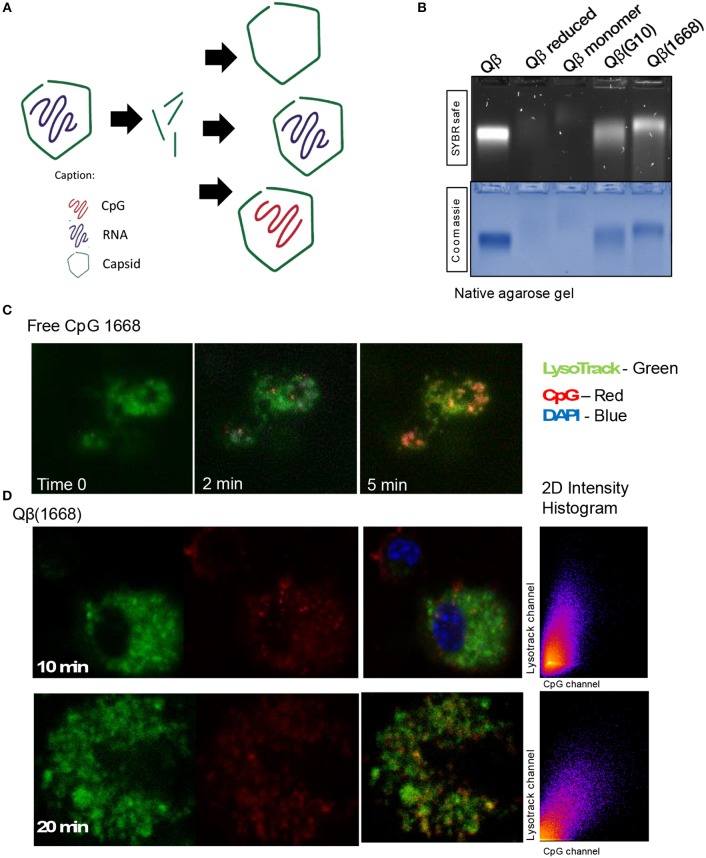
Packaging CpGs into VLPs alters endosomal trafficking of CpG. **(A)** Schematic representation of re-packaging of VLPs with polyanionic molecules. **(B)** Re-packaging of VLPs analyzed by co-localization of protein and nucleic acid in a native agarose gel stained with SYBR safe for nucleic acid (upper panel) and Coomassie for proteins (lower panel). First lane shows the migration pattern of a purified and intact VLP packaging RNA. Lane 2 and 3 with samples from the reduced and disassembled VLP. 4 and 5 shows the re-assembled VLPs with A type CpG and B type CpG. **(C)** Time-lapse of *in vitro* uptake of free CpG 1668 by BMDCs. Lysosomes is represented in green (GFP), CpG in red (Alexa Fluor 567). **(D)** Endosomal trafficking of B type CpG packed into Qβ VLP. BMDCs were pulsed with the VLP for 10 min (upper panel) or 20 min (bottom panel), washed and immediately fixed. First image in green shows the endosomal compartment, followed by the CpG channel. Third image contains the superposition of both channels (plus DAPI stain for the nuclei). 2D intensity histogram showing pixel co-localization of the two channels. Data is representative of 3 independent experiments.

It has been previously described that CpGs associated with liposomes trigger distinctive immune responses when compared to free molecules due to distinct spatial-temporal distribution of the CpG/liposome, which dictates the interaction of CpGs with components of the signaling cascade of TLRs (22). To investigate whether the same phenomena would be influencing the cytokine production observed here, a time-lapse experiment of CpG uptake by BMDCs was performed by pulsing cells with the type B CpG 1668 containing a fluorescent dye. We found that free CpG co-localizes with the lysosomal marker LysoTrack as fast as 6 min after addition of CpG to the cells (Figure 3C). Next, the spatiotemporal distribution of Qβ(1668) was tested in a similar setting. To that end, BMDCs were pulsed with the VLP containing CpG coupled to a fluorescent tag, washed, and fixed at the indicated time points (Figure 3D). Co-localization of packaged CpG and LysoTrack dye was delayed in comparison to the free CpG. At the 10 min time-point (Figure 3D upper panel), no co-localization was observed between CpG and the LysoTrack dye, as evidenced in the 2D intensity histogram of the co-localization of pixels in each of the two analyzed channels. Only at a later time-point (20 min), extensive co-localization could be detected, demonstrating that CpG reach the lysosomal compartment in a delayed fashion if packaged into VLPs. Thus, packaging CpG into VLP alters their endosomal trafficking, which may explain the distinct immune responses induced in DCs.

### Qβ Packaging Type B CpG Induces Superior Antigen-Specific CD8+ T Cell Responses Compared to Qβ Packaging RNA

It has been consistently shown in the literature that VLP-based vaccines containing CpG are able to induce strong immune responses and enhance protection in different challenge models. For instance, Qβ(1668) coupled to a LCMV-derived peptide or HPV-derived E7 protein had shown excellent protection in viral (5) and tumor models (8), respectively. The consistently superior ability of Qβ(1668) in comparison to Qβ(RNA) to induce protection in different models in a CTL-dependent manner propelled us to further investigate the initial events in the immune response that were leading to appropriate activation of T cells.

**Figure 4:**
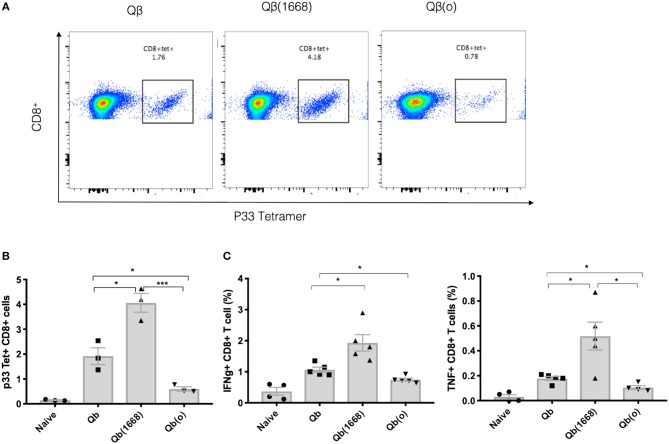
Packaging of CpGs on VLP alters the immune response induced by VLPs. C57BL/6 mice were vaccinated s.c. with 50 μg of VLP-p33 containing RNA (*E. coli*), CpG class B or the empty capsid. **(A,B)** p33-specific tetramer staining of CD8^+^ T cells from 3 immunized mice and **(C)** ICS staining of CD3^+^CD8^+^ T cells 7 days post vaccination. 6 h stimulation with p33 peptide from 5 immunized mice. Data represented as Mean ± SEM. Statistical significance was measured by unpaired two tailed *t-*tests followed by Bonferroni-Dunn's correction for multiple testing. ^*^p < 0.01, ^***^p < 0.0009. Data is representative of 3 independent experiments.

To demonstrate the distinct induction of T cell responses promoted by type B CpG (TLR9 ligand), RNA (TLR7/8 ligand) or in absence of TLR stimulation *in vivo*, Qβ(1668), VLPs containing prokaryotic RNA [represented as Qβ(RNA)], and VLP devoid of nucleic acid [as Qβ(o)] were cross-linked to the immunodominant LCMV-derived peptide gp33_33−41_ and mice were immunized via the s.c. route. The antigen specific CTL response was evaluated 7 days after immunization (Figures 4A,B). Packaging of type B CpG into VLPs had not only increased the expansion of p33-specific T CD8^+^ cells as previously demonstrated by tetramer staining (Figure 1B) but had also boosted the amount of key cytokines such as IFN-γ and TNF-α being produced in response to antigen stimulation (Figure 4C). Qβ(RNA) also induced antigen-specific T cell responses and cytokine production but to a much lesser extent when compared to Qβ(1668). VLPs devoid of nucleic acids, on the other hand, induced responses comparable to naïve mice. Additionally, mice lacking cytoplasmic nuclei-acid sensors stimulator of interferon genes (STING, also known as Myps and TMEM173), the adaptor molecule of the retinoic acid-inducible gene-I-like receptors (RIG-I) and mitochondrial antiviral protein (MAVS) were immunized following the same regimen and no statistically significant differences were observed in T cell responses, ruling out a role for such sensors in T cell responses induced by VLPs loaded with TLR7/8 or 9 ligands (Figure S1).

### Transcriptional Signature in Splenic DCs 24 h Post Immunization With VLPs Containing TLR9 or TLR7 Ligands as Adjuvants

To evaluate the impact of the nucleic acid packaged within the VLP on the innate immune response, mice were immunized with 50 μg of the respective VLP and 24 h later spleens were collected and processed for analysis. Total RNA was extracted from sorted CD11c^+^ murine splenic cells. Following rigorous quality control, RNA samples were analyzed by genome-wide RNA-sequencing.

Mice immunized with VLP devoid of TLR ligands were used as control for the groups receiving VLPs associated with TLR ligands. The baseline normalized log2 gene expression values for genes with a significant change in expression between groups (adjusted *P* < 0.05 FDR) were classified as differentially expressed genes (DEGs). The differential expression of these genes was analyzed for statistical significance by one-way analysis of variance (ANOVA).

**Figure 5:**
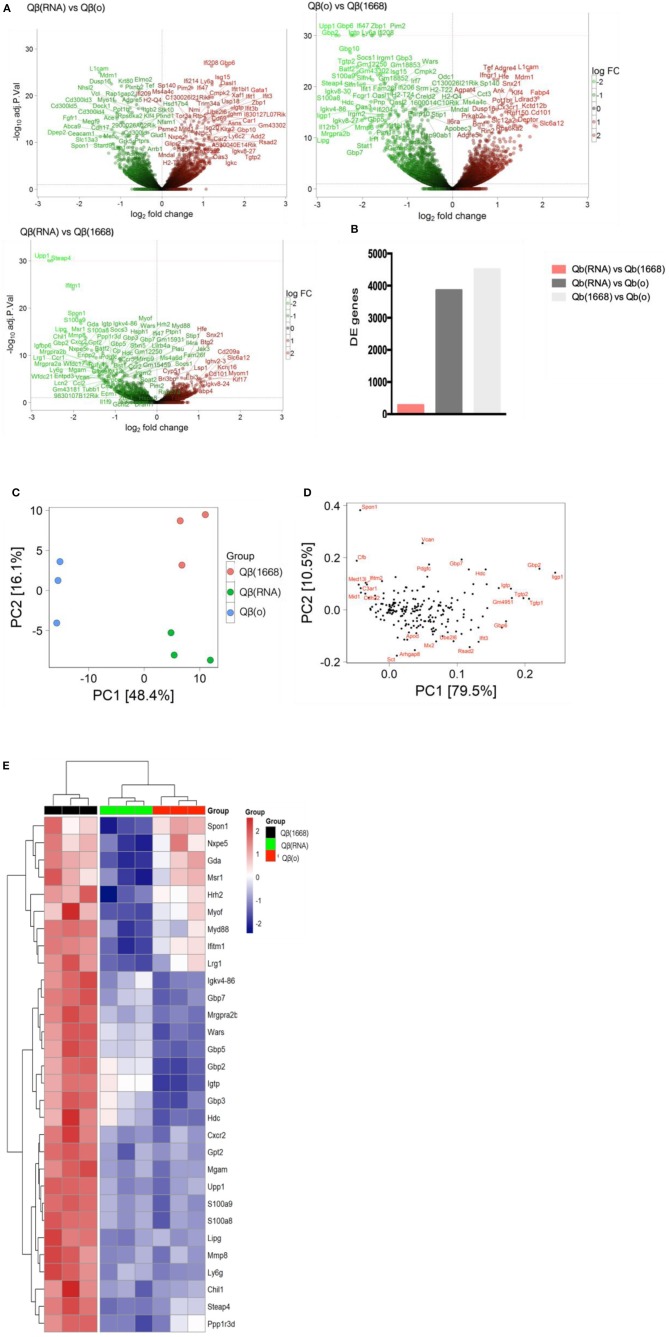
Transcriptional reprograming of DCs 24 h after immunization. **(A)** Volcano plot of genes differently expressed between Qβ(RNA) vs. Qβ(o), Qβ(o) vs. Qβ(1668), and Qβ(RNA) vs. Qβ(1668), respectively. Log2 fold change on the x-axis and the -Log10 *p*-values on y-axis. Up-regulated genes are represented in red and down-regulated gene in green. **(B)** Number of genes differently expressed between the groups with a FDR of 10%. **(C)** Principal component analysis of genes differently expressed among the groups. The effects of the principal component 1 (PC1) is shown on x-axis and principal component 2 (PC2) shown in the y-axis **(D)** Genes with major contribution to sample clustering. **(E)** top 30 differently expressed genes. Hierarchical clustering of top 30 differently expressed genes. Regularized log transformed count data were row scaled and hierarchical clustering was applied to Euclidean distances using complete linkage clustering.

VLPs loaded with a TLR-ligands (CpG 1668 or RNA) led to differential expression of more than 3,800 genes in comparison to the formulation devoid of TLR (3,855 genes between Qβ(1668) vs. Qβ(o) and 4,504 genes between Qβ(RNA) vs. Qβ(o), showing the major transcriptional changes that TLR engagement promotes on the cell, while Qβ(RNA) vs. Qβ(1668) had far fewer genes differently expressed, with 279 genes in total (Figures 5A,B).

Later, aiming to assist the characterization and understanding of the regulation of genes controlling the immune response, an unsupervised principal component analysis (PCA) was performed on the data set and revealed an expected clustering of samples according to the type of PRR ligand packaged within the VLP (Figures 5C,D). Considering that the three groups had received the same VLP packaging different TLR-ligands, the genes most significantly contributing to the clustering of the samples (Figures 5C,D) can be attributed to the distinct transcriptional signature triggered by the TLR ligands and not the VLP itself.

The top 30 differently expressed genes between the groups were analyzed for hierarchical clustering performed by calculating the Euclidean distances using complete linkage clustering method. Figure 5E shows the top 30 genes differently expressed between Qβ(1668) vs. Qβ(o). Clustering analysis showed a distinct expression profile between the three groups with genes mostly involved in immune responses and TLR signaling such as the gene coding for the adaptor molecule Myd88, the alarmins S100a8/9 and members of the family guanylate binding proteins (GBPs). In addition to genes involved in immune responses, several genes responsible for controlling cell homeostasis, cell signaling, and metabolism were also differently expressed. Examples of note were the Steap4 and MMP8 genes, two metalloreductase that have been shown to integrate metabolism and inflammatory responses (23), and Upp1 (24) an enzyme that catalyzes events related to transcription and translation of proteins. It is noteworthy that the major transcriptional changes that DCs undergo upon TLR engagement are related to either metabolism or immune responses. This a direct consequence of the metabolic program that DCs need to undergo in order to support the immune response, gene transcription and translation, protein and cytokines production, and cell migration, which represent major energetic demands for the cell (25). Surprisingly, Qβ(RNA) clustered closer to Qβ(o) compared to Qβ(1668), showing the top 30 genes differently regulated in response to CpG is quite distinct from RNA-induced responses, despite engagement of TLRs both driving MyD88-dependent responses.

### Functional Analysis of Transcriptional Signature Reveals Important Pathways Engaged by the Qβ VLP Containing TLR Ligands

Following the initial characterization of the transcriptional regulation, additional data mining methods were employed to facilitate the identification of patterns of gene expression at the system level. Functional enrichment analysis of genes differently expressed was performed using the ClusterProfile method (26, 27) revealing important immune pathways and genes that were differentially regulated across the groups. Qβ(1668) and Qβ(RNA) in comparison to Qβ(o) up-regulated pathways related to immune system or metabolism, such as cytokine signaling and biosynthesis of amino acids, respectively (Figure S2).

**Figure 6:**
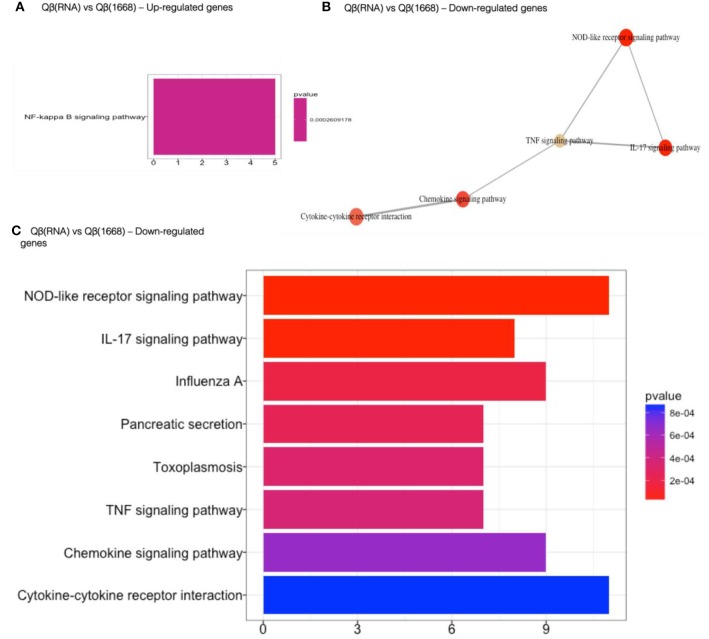
Gene set enrichment analysis of differently regulated genes on Qβ(RNA) vs Qβ(1668). **(A)** Gene set enrichment analysis of up-regulated genes). **(B)** Gene set enrichment analysis of down-regulated genes. Pathways sharing gene sets are connected by a line. **(C)** Gene set enrichment analysis of down-regulated genes. Significance displayed as a color scale as indicated by the *p*-value. X-axis: number of genes in the dataset per pathway.

Qβ(1668) in comparison to Qβ(RNA)-recipient group (Figure 6) showed that among the 279 differently expressed genes, relevant immune pathways such as chemokine signaling pathway and cytokine-cytokine receptor interaction were enriched. In addition to those general pathways, genes belonging to the NOD-like receptor pathway such as NLRP12, p38, GBPs, CCL2, and CCL12 were significantly upregulated by Qβ(1668). Other pathways involving cytokine and chemokine signaling and production were also up-regulated, including TNF-α and IL-17 pathways. Regarding the genes up-regulated by Qβ(RNA), there was a set of genes belonging to the anti-apoptotic NFκ-B pathway such as Bcl2a1d (28). We have also performed KEGG module pathways analysis on genes downregulated on the Qβ(RNA) vs. Qβ(1668), showing the enrichment of genes belonging to the modules ECS complex, JAK-STAT signaling, and Acylglycerol degradation. The complete list of differently regulated pathways and the genes contributing to the enrichment of pathways in the comparison of Qβ(RNA) vs. Qβ(1668), are listed in Table 1.

**Table 1:** Enriched pathways on Qβ(RNA) vs. Qβ(1668).

**Description**	**Gene ratio**	**Bg ratio**	***p*-value**	***p*. adjust**	***q*-value**	**Gene name**	**Count**	**FC**
NF-kappa B signaling pathway	5/46	104/8263	0.00026092	0.02530903	0.02361993	Relb/Bcl2a1a/Bcl2a1b/Gadd45b/Bcl2a1d	5	Up
NOD-like receptor signaling pathway	11/100	168/8263	5.2401E-06	0.00101658	0.00088806	Gbp2/gbp7/nlrp12/CCL2/Gb3/CCL12/Mapk13/Myd88/Oas2/Hsp90ab1	11	Down
IL-17 signaling pathway	8/100	91/8263	1.2895E-05	0.00125084	0.00109271	S100a9/S100a8/CCL2/Lcn2/CCL12/Mmp9/Mapk13/Hsp90ab1	8	Down
Influenza A	9/100	168/8263	0.00018687	0.01144463	0.00999775	CCL2/SOCS3/CCL12/22074/Try4/Mapk13/Myd88/Furin/Oas2/Hspa8	9	Down
Pancreatic secretion	7/100	103/8263	0.0002361	0.01144463	0.00999775	Ctrl/Pnliprp1/Amy2b/Try4/Cpa2/Bst1/Atp2a2	7	Down
Toxoplasmosis	7/100	108/8263	0.00031631	0.01144463	0.00999775	Igtp/SOCS1/Maok13/CCR5/Irgm1/MyD88/Hspa8	7	Down
TNF signaling pathway	7/100	110/8263	0.00035396	0.01144463	0.00999775	CCL2/SOCC3/CCL12/Mmp9/Mapk13/Irf1/Ifi47	7	Down
Chemokine signaling pathway	9/100	199/8263	0.0006522	0.01807516	0.01579002	CXCR2/CXCR1/CCL2/Pppb/CCR1/CCL12/CCR5/Jak3/Nras	9	Down
Cytokine-cytokine receptor interaction	11/100	296/8263	0.00085723	0.02078771	0.01815964	CXCR2/CXCR1/CCL2/Ppbp/IL-1f9/CCR1/CCL12/CCR5/IL12rb1/Csf3r/16190/IL4ra	11	Down
Prolactin signaling pathway	5/100	72/8263	0.00173004	0.03729202	0.0325774	SOCS3/SOCS1/Mapk13/Irf1/Nras	5	Down

### Pro-inflammatory and Anti-inflammatory Response Are Tightly Regulated in the Early Stages of Response

DCs undergo a complex maturation process that involves upregulation of MHC and co-stimulatory molecules. The activation events are accompanied by the secretion of cytokines that will act in an autocrine and paracrine manner impacting not only the DC‘s phenotype, but most immune cells equipped with the respective receptors as well. Considering that most of the pathways differently expressed between Qβ(RNA) and Qβ(1668) were related to immune responses triggered by cytokines, the subsequent analysis was focused on assessing cytokine production.

**Figure 7:**
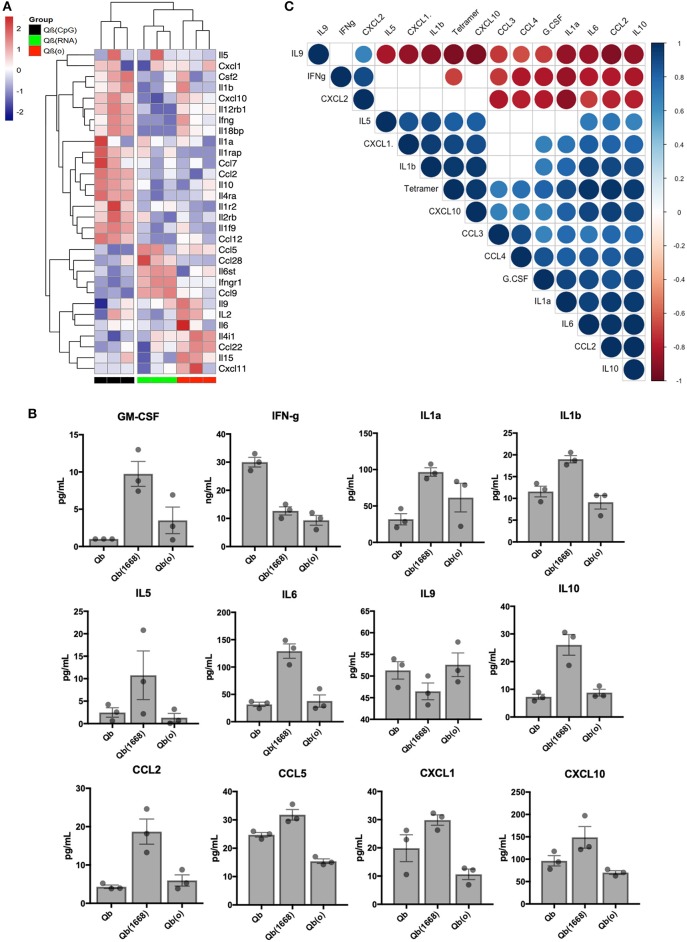
Immunization with the different VLPs leads to distinct Cytokines production. **(A)** Expression data (multi mapped counts per million) of selected cytokines 24 h post immunization in splenic DCs (+12 h incubation). Hierarchical clustering of top 30 differently expressed cytokines. Clustering calculated based on the Euclidean distances using complete linkage clustering. **(B)** Secreted cytokines by splenocytes 24 h post immunization. Values represented as mean + SEM. *N* = 3 mice. **(C)** Correlation matrix of cytokines secreted by splenocytes 24 h post immunization. Positive correlations are displayed in blue and negative correlations in red color. Color intensity and the size of the circle are proportional to the correlation coefficients. Legend colors shows the correlation coefficients and the corresponding colors. Correlation calculated using Pearsons coefficient. Correlations with significance *p* > 0.01 are depicted in white.

Aiming to have a better understanding of the cytokines being differentially regulated in response to immunization, we first analyzed RNA expression levels for cytokines in detail (Figure 7A). Based on the 30 selected cytokines the resulting clustering analysis of the expression level of each cytokine displayed an expression pattern correlated to the type of TLR ligand packaged in the VLP given as a vaccine. The group receiving Qβ(1668) up-regulated expression of several pro-inflammatory cytokines such as INF-γ, IL-2, and several chemokine receptors as well as IL-1R, IL-12R, whilst down-regulating IL-4, a cytokine known to promote Th2 polarization. The group receiving Qβ(RNA) clustered closer to Qβ(o) instead of Qβ(1668). Differences were especially seen for the cytokines IL-10, CCL2, and IL-1 to name a few.

In order to validate the mRNA expression of cytokines, key cytokines secreted 24 h following *in vivo* immunization were measured by Milliplex (Figure 7B). Mice were immunized following the same regimen stated previously, 24 h later splenocytes were collected and incubated for further 12 h. Qβ(1668) induced higher levels of secretion of several inflammatory and some anti-inflammatory cytokines. As expected, most of the cytokines followed the same trend as seen in the RNAseq data. As examples may serve pro-inflammatory cytokines and chemokines, such as IL1-α, IL1-β, IL-6, and CCL2, and IL-10 as anti-inflammatory cytokine. The Th9-associated cytokine IL-9 on the other hand was produced in higher levels by the Qβ(RNA) and Qβ(o) immunized groups, corresponding to the RNA expression data and showing the dominating Th1-biased response induced by CpG 1668. Contrary to the RNA expression data, IFN-γ was mostly produced in response to Qβ containing RNA, which could be an indication that there are other cells than DCs producing IFN-γ in response to the RNA compared to CpG.

Aiming to identify cytokines produced 24 h post immunization that could be correlated to T cell responses, a pairwise correlation analysis of the average cytokine production and outcome of T cell responses as measured by tetramer staining was performed. The correlation matrix (Figure 7C) showed that the tested variable of tetramer staining was positively correlated to IL-6, IL-10, CXCL10, CCL2, IL-1a, IL-1b, CXCL1, and TNF-α. The cytokine IL-9 and IFN-γ were negatively correlated to tetramer staining while the chemokines CXCL2, CCL3, CCL4, and growth factor G-CSF did not reach statistical significance (*p* < 0.01). Of note, cytokines IL-2, IL-4, IL-7, IL-12p40, and IL-12p70 and IL-15 had also been measured but were below the detection limit of the assay.

Interestingly, type B CpG had been consistently described as an inducer of IL-12 secretion by us and others (29), namely when testing in an *in vitro* setting. In our *in vivo* experimental setting, however, we were unable to detect IL-12 at this time point. We have also observed a higher production level of GM-CSF in the Qβ(1668) receiving group, a cytokine that is critical for DC activation and is typically produced by naïve T cells and NK cells (30). These data underpin the complexity of the orchestration of T cell responses in response to vaccines and the complex cross-modulation of innate and adaptive immune system.

### CCL2 Modulates CD8+ T Cell Responses Induced by Qβ(CpG)

**Figure 8:**
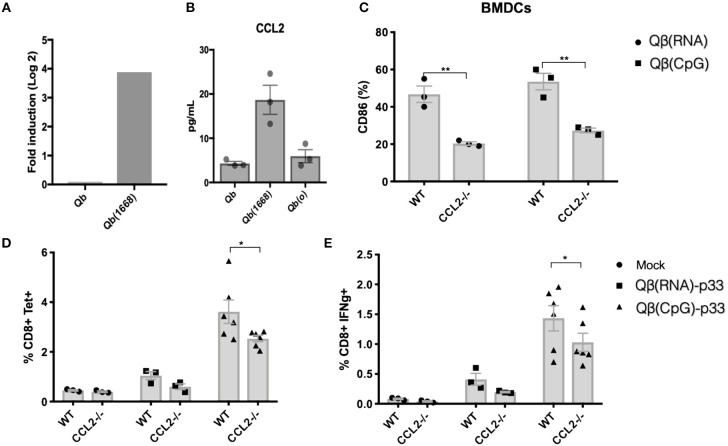
CCL2 is up-regulated in DCs immunized with Qβ(1668) that modulates CTL responses. **(A)** CCL2 mRNA expression 24 h following immunization with Qβ(1668) or Qβ in comparison to Qβ(o). Data is represented as mean fold change. *N* = 3 mice per group. **(B)** CCL2 secreted in supernatants of splenocytes isolated after 24 h of immunization and 12 h of incubation *in vitro*. *N* = 3 mice per group. **(C)** CD86 expression on BMDCs pulsed with VLPs for 3 h p33-specific tetramer staining of CD8+ T cells. BMDCs were generated from pooled bone marrow from 2 mice. 3 individual replicates per condition. **(D)** Tetramer staining of CD8+ T cells 7 days post immunization. **(E)** ICS staining of CD3^+^CD8^+^ T cells 7 days post vaccination. 6 h stimulation with p33 peptide. Data represented as Mean + SEM of 2 combined experiments. Statistical analysis was performed by calculating the Z score of the combined *p*-values of the two independent experiments. ^*^p < 0.04, ^**^p < 0.02.

Considering the evident up-regulation of CCL2 mRNA on DCs, the higher levels of CCL2 in the supernatant of splenocytes from mice immunized with Qβ(1668) (Figures 8A,B, respectively) and the positive correlation of CCL2 production and T cells responses (Figure 7C) we sought to investigate the role of CCL2 in CTL responses *in vivo*.

First, we investigated upregulation of activation makers in BMDCs from CCL2−/− vs. control mice in response to VLPs. CD86 upregulation was reduced in response to both Qβ(RNA) and Qβ(1668). To evaluate the impact of CCL2 on CTL responses, sex and age matched CCL2−/− mice and WT counterparts were immunized with Qβ(RNA)-p33 or Qβ(1668)-p33 and T cell responses were subsequently assessed 7 days later. CCL2−/− mice showed a reduction in the clonal expansion of p33 specific T cells (Figure 8). In addition to that, the capacity of those cells to produce IFN-γ was reduced compared to the WT (Figure 8E). The reduced response likely reflects a modulatory effect of CCL2 on the T cell response.

## Discussion

In the present study, the modulation of the innate immune response mediated by VLPs packaging TLR ligands was investigated and characterized in the context of vaccines. The outcome of immunizations employing VLPs loaded with PRR ligands was evaluated and correlated to the early events in DCs orchestrating subsequent adaptive responses mediated by T cells.

We show here that packaging CpGs into VLP alters their endosomal trafficking. Packaged CpG is retained for longer periods of time in endosomal compartments before reaching lysosomes, which may impact the immune response as seen by differential cytokine production induced by free and packaged versions of CpGs.

The initial innate immune response mediated by DCs influences subsequent adaptive immune responses; here, the early transcriptional signature in DCs in response to immunization was characterized and we correlated these findings to the observed T cell responses. Functional analysis of the genes expressed in response to the different VLP formulations indicated important immune pathways being differently regulated 24 h post immunization.

From those pathways, CCL2 was selected for further investigation due to its markedly distinct expression in response to CpG and its relevance to the overall immune response. We found that CCL2 chemokine produced by DCs 24 h after immunization onwards modulates the quality of the cytokines produced by T cells in a later stage of the response. CCL2 has been shown to support DC-maturation (31) and the recruitment and modulation of IFN-γ production by T cells (32). The immune response seen in CCL2−/− mice was only slightly reduced, and observation may be explained by the redundancy of the chemokine system. CCL2 signals through CCR2, a receptor shared with several other chemokines; lack of CCL2 may therefore have been partly compensated by re-modeling of the milieu of chemokines that maintains T cell responses. In addition to CCL2, other chemokines and cytokines were positively correlated to CTL responses in our model, including pro-inflammatory cytokines and also IL-10, a cytokine known for its anti-inflammatory activity on T cells. IL-10 is traditionally considered as a negative regulator of protective immune responses. Our data here support recent findings indicating a more multifaceted behavior of IL-10 (33). IL-10 analyzed in the complex cytokine milieu induced *in vivo* seems to support CTL responses, by either modulating the response to avoid excessive immune activation or yet, by controlling suppressive subsets of CD4+ T cells (34). Alternatively, IL-10 may be induced by pro-inflammatory cytokines that enhance CTL-responses and IL-10 may not causally linked to enhanced CTL responses but merely reflect the presence of these other cytokines. Finally, we found no role for STING or MAVS in the VLP-induced CTL responses.

In summary, this work contributes to the understanding of DC-mediated immune responses in the context of vaccines and their development and further establishes the potential of VLPs to modulate the immune response. Finally, our data support the notion that immune responses are orchestrated by a complex milieu of cytokines and immune cells and that there are several processes occurring in parallel that shape and direct the overall response. Understanding such processes is a challenging but rewarding endeavor as many therapeutic clues and basic knowledge can be derived from research of this kind. Moreover, VLPs and CpGs are currently being employed for clinical use, especially in vaccine formulation; we hope to contribute to the understanding of the immune activity of both compounds and facilitate their use as vaccines.

## Data Availability

The RNAseq data generated for this study can be found in the Gene Expression Omnibus (GEO), under the accession code GSE134192. Additional datasets may be available upon reasonable request.

## Ethics Statement

This study was carried out in accordance to the recommendations of the Animals (Scientific Procedures) Act 1986 (ASPA) and European Directive 2010/63/EU on the protection of animals used for scientific purposes. Protocols were approved by the Animal Welfare and Ethical Review Bodies at the Nuffield Department of Clinical Medicine, University of Oxford (PPL 30/2947).

## Author Contributions

MB and AG designed the experiments. AG, MM, FL, and GC-M performed the experiments. AG and JM analyzed the data. MB and AG had written the manuscript.

### Conflict of Interest Statement

The authors declare that the research was conducted in the absence of any commercial or financial relationships that could be construed as a potential conflict of interest.

## Supplementary Material

Figure S1 STING −/−, MAVS −/−, and age and sex matched C57BL/6 wild type mice were vaccinated s.c. with 50 μg of VLP-p33 **(A)** p33-specific tetramer staining of CD8^+^ T cells. **(B,C)** ICS staining of CD3^+^CD8^+^ T cells 7 days post vaccination. 6 h stimulation with p33 peptide. Data represented as Mean ± SEM of *n* = 3 mice/group. Statistical significance was measured by unpaired two tailed *t-*tests followed by Bonferroni-Dunn's correction for multiple testing. ^*^*p* < 0.01, ^***^*p* < 0.0009. Data is representative of 3 independent experiments.

Figure S2 Gene Ontology of genes belonging to the sub-ontology “Biological Processes.” Genes differently expressed between DCs from immunized mice receiving **(A)** Qβ(RNA) vs. Qβ(o) and **(B)** Qβ(o) vs. Qβ(1668), and **(C)** Qβ(RNA) vs. Qβ(1668). –log 10 of q values. % of DX: percentage of genes differently expressed. Data was analyzed using the package DAVID for GO analysis.
