# Supplementary figures and images for: Early Transcriptional Signature in Dendritic Cells and the Induction of Protective T Cell Responses Upon Immunization With VLPs Containing TLR Ligands—A Role for CCL2
Source: Front Immunol. 2019 Aug 2;10:1679. doi: 10.3389/fimmu.2019.01679 (PMC6687836; doi:10.3389/fimmu.2019.01679)

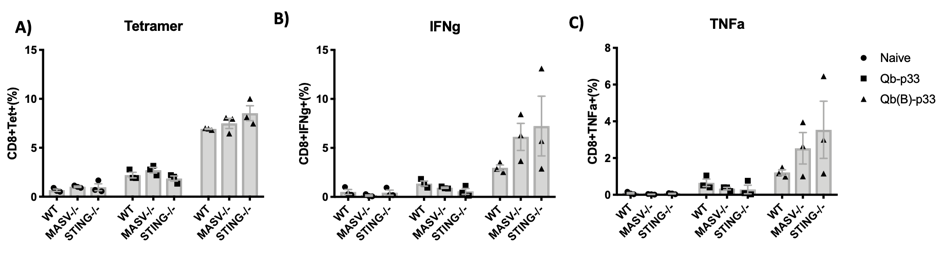

Supplement: Figure S1 — STING −/−, MAVS −/−, and age and sex matched C57BL/6 wild type mice were vaccinated s.c. with 50 μg of VLP-p33 (A) p33-specific tetramer staining of CD8+ T cells. (B,C) ICS staining of CD3+CD8+ T cells 7 days post vaccination. 6 h stimulation with p33 peptide. Data represented as Mean ± SEM of n = 3 mice/group. Statistical significance was measured by unpaired two tailed t-tests followed by Bonferroni-Dunn's correction for multiple testing. *p < 0.01, ***p < 0.0009. Data is representative of 3 independent experiments. [file Image_1.PNG]

## A) Q $\beta$ (RNA) vs Q $\beta$ (o) – 4503 genes

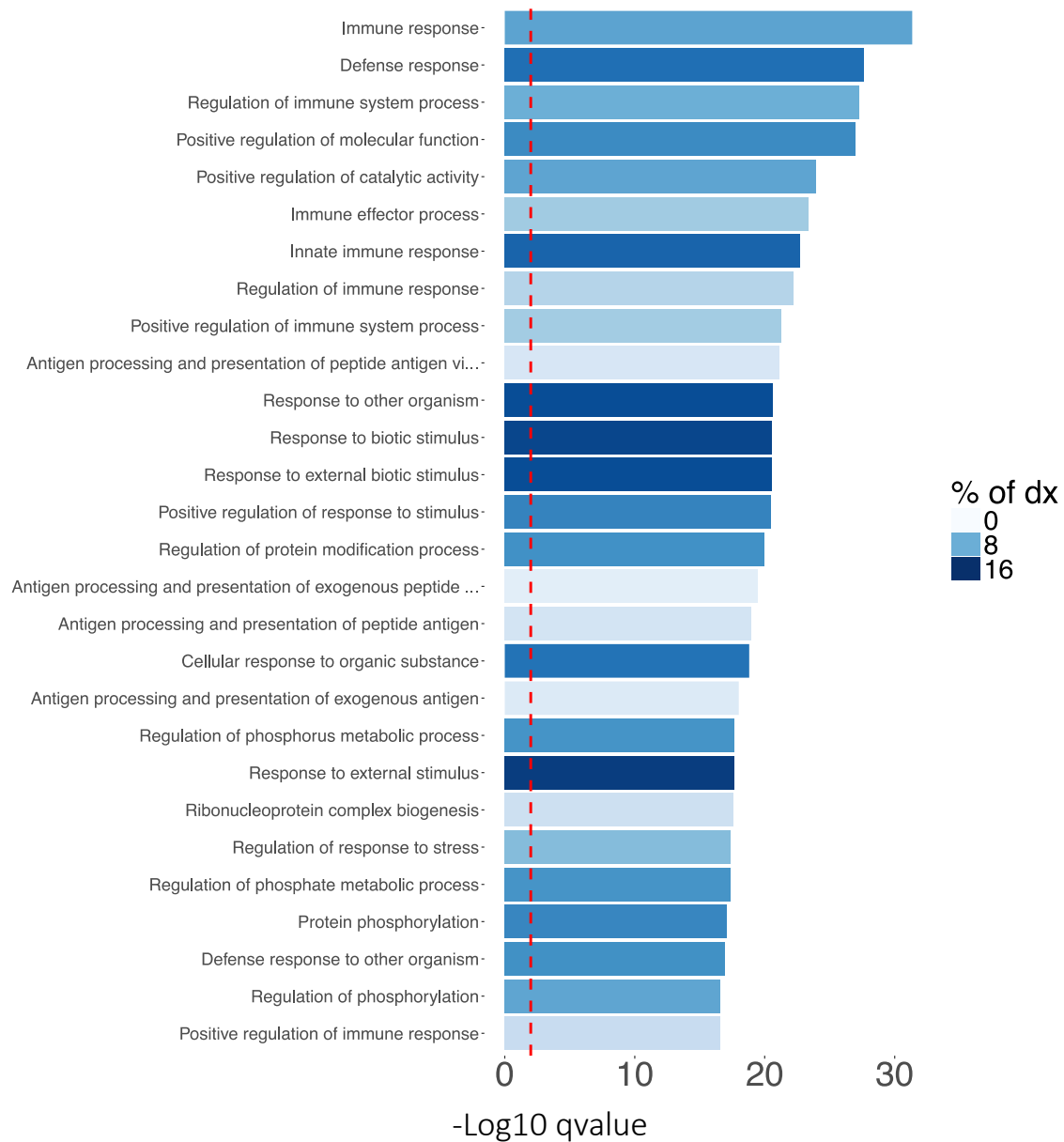

## B) Q $\beta$ (o) vs Q $\beta$ (1668) - 3853

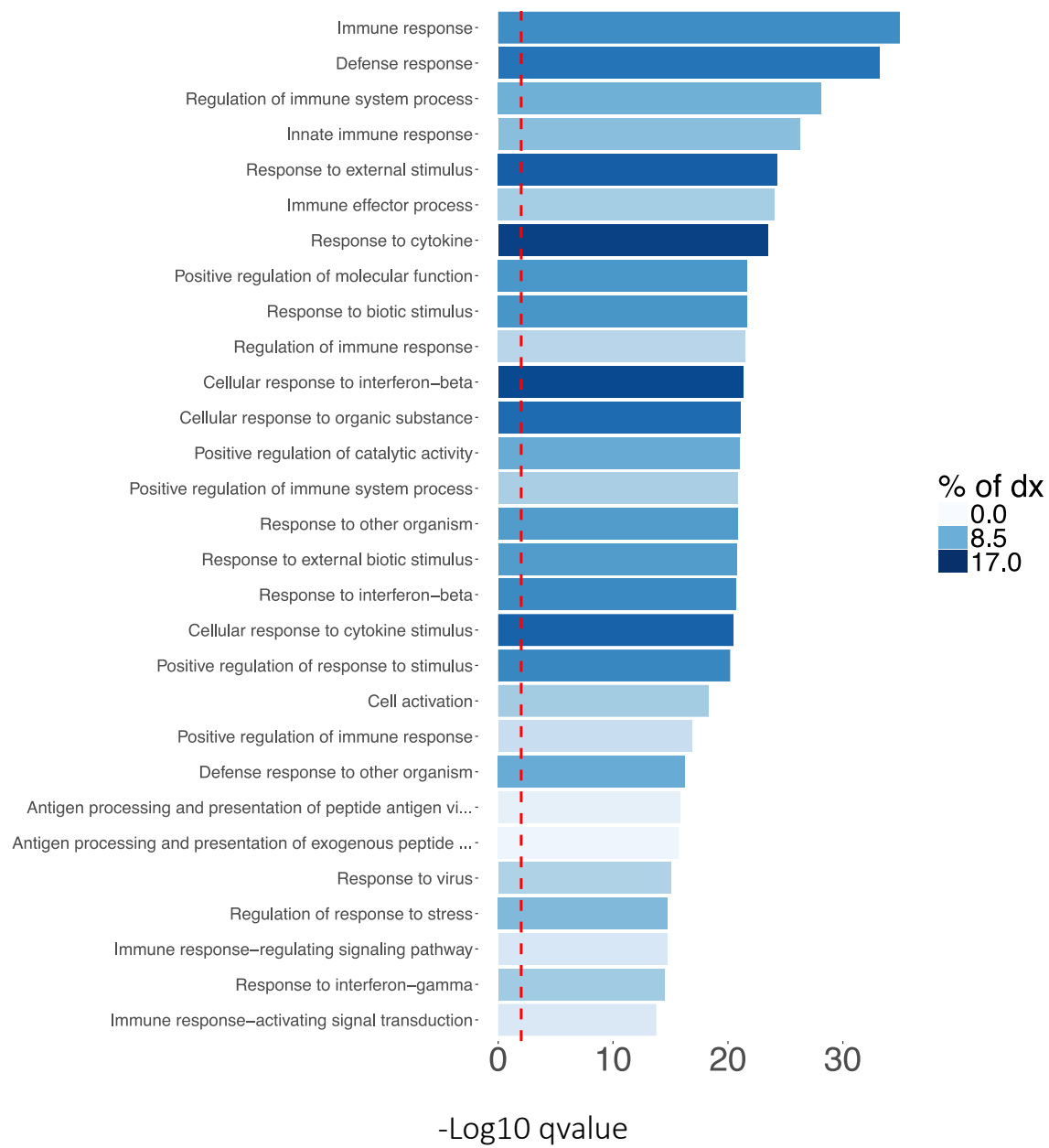

### C) Q $\beta$ (RNA) vs Q $\beta$ (1668) – 279 genes

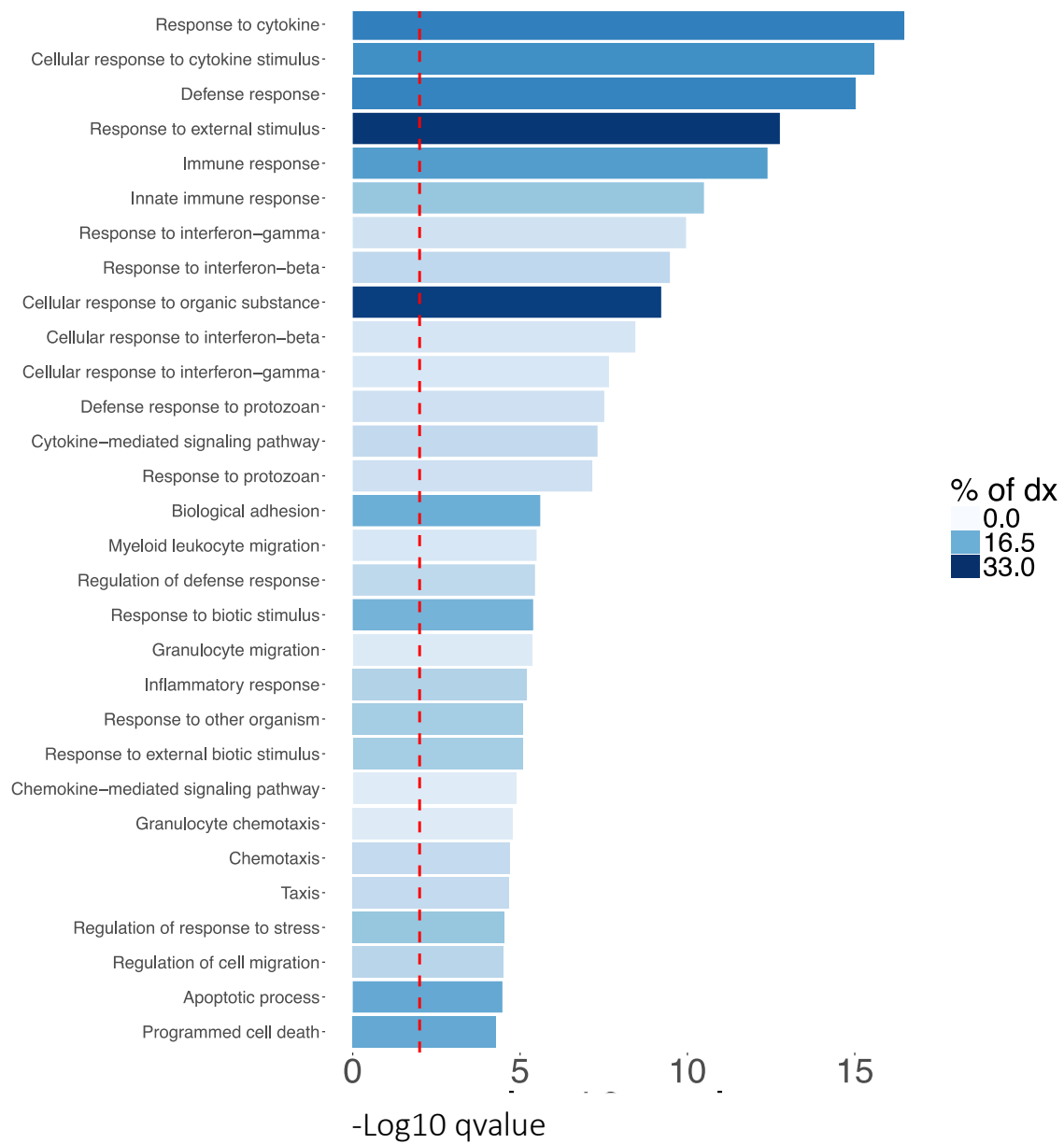

Supplement: Figure S2 — Gene Ontology of genes belonging to the sub-ontology “Biological Processes.” Genes differently expressed between DCs from immunized mice receiving (A) Qβ(RNA) vs. Qβ(o) and (B) Qβ(o) vs. Qβ(1668), and (C) Qβ(RNA) vs. Qβ(1668). –log 10 of q values. % of DX: percentage of genes differently expressed. Data was analyzed using the package DAVID for GO analysis. [file Image_2.pdf]
